# Converging arboviral outbreaks demand disease-agnostic public health responses

**DOI:** 10.1016/j.ebiom.2026.106519

**Published:** 2026-10-14

**Authors:** eBioMedicine

The scale of the 2023–25 Oropouche virus (OROV) outbreak in Brazil was itself unprecedented, and coincided with a record-breaking 2023–24 dengue epidemic, presenting challenges in diagnosing and tracking both. In name and perception, arthropod-borne virus (arbovirus) outbreaks are often considered separate and distinct, yet nature never neatly compartmentalises, and the same meteorological and ecological factors that drive one can affect many. Expanding insect-vector habitats increasingly allows arboviruses to stably co-circulate in overlapping regions. Yet, despite advances in the development of pathogen-specific diagnostic tools, many acute febrile illnesses, particularly in low-income and middle-income countries, remain without a confirmed aetiological diagnosis.

Early dengue and Oropouche disease, much like many arboviral infections, are difficult to distinguish clinically, sharing symptoms such as fever and headache; however, prognoses can differ considerably. Severe dengue is characterised by internal bleeding and organ impairment, whereas Oropouche fever can progress to neurological disease in adults and can cause congenital abnormalities during fetal brain development. Recent mechanistic studies, including work published in *eBioMedicine*, have begun to elucidate the pathways underlying Oropouche-associated neurological disease, and have shown the effect of OROV infection on neurodevelopment in human neural organoids.

Misclassification is not merely an epidemiological challenge. Interventions, diagnostics, surveillance systems, and funding mechanisms are still predominantly organised around individual pathogens, and so can be outpaced by the spread of a previously overlooked virus. Public health systems cannot reliably predict what the next outbreak will be, and so arbovirus outbreak preparedness must be disease-agnostic.

In Singapore, a large cluster-randomised controlled trial involving over 700 000 people showed the effects of highly controlled releases of *Wolbachia* bacteria-infected *Aedes aegypti* mosquitoes. These laboratory-grown male mosquitoes mate with wild females but produce no viable offspring, suppressing mosquito populations and thereby inhibiting viral transmission. Analysis published in the October, 2026, issue of *eBioMedicine* showed that local epidemiologically linked dengue incidence was reduced by 61–99% over the 24 months following these releases. Similar programmes are beginning to be rolled out against dengue in Mexico, Brazil, and Laos, and by Google’s ‘Debug’ project in the USA.

A crucial advantage of vector-targeted interventions is that they remain effective even when surveillance hasn’t identified all circulating pathogens. *A. aegypti* mosquitoes are also vectors for chikungunya, Zika, and yellow fever viruses, so the benefits of targeting them might extend well beyond dengue. A commentary linked to this trial further highlights how synergism with vaccine efforts and impact on other arboviruses remain largely unknown. Likewise, the effects of geographical spillover and vacant ecological niches on populations of alternative arboviral vector mosquito species remains under intense scrutiny. So, in the era of arboviral convergence, diagnostics too must go further than single-pathogen or single-vector approaches, and must instead be sensitive to a widening range of possible infections.

RT-PCR-based panels offer a well-tested and modular method to distinguish a broad range of early arbovirus infections and other febrile illnesses within the initial viraemic phase; however, these require specialised and centralised equipment that are lacking in rural, under-resourced areas. Serological tests are useful for establishing previous or later-stage infections, but multiplex rapid diagnostic tests deployed at the point of care are crucial during active arboviral outbreaks, enabling timely clinical decisions including improved triage and the discontinuation of unnecessary antibiotics. Rapid diagnostic tests that detect a small subset of arbovirus antigens (dengue, chikungunya, and Zika viruses) and antibodies against them remain under early evaluation, while even singleplex point-of-care tests for Oropouche virus (such as RT-LAMP) are yet to be clinically deployed. Ultimately, competent outbreak preparedness will require syndromic diagnostic platforms capable of distinguishing between multiple arboviruses within a single clinical encounter.

Thus, even within well-established disease-specific monitoring systems, clinically similar but unexpected arboviruses can circulate undetected. In tropical Sri Lanka, metagenomic analyses identified cryptic chikungunya virus infections among patients initially diagnosed with dengue. In more temperate regions, Italy and France have reported locally acquired dengue cases, and West Nile Virus transmission is now established throughout southern Europe, with cases reported as far north as Germany. In London, the first evidence that *Aedes aegypti* mosquitoes were breeding within the UK was reported on Sept 3, 2026.

There is much to be learnt from the tropical regions where arboviruses have long been endemic, and so these major overlapping outbreaks must serve as a warning. As the ecological barriers that historically separated arboviruses become less reliable, fewer regions are insulated from the co-circulation of multiple arboviral threats. Preparation against converging, rather than emerging, arboviruses must now be considered globally.

